# Antifungal Activity of Chitosan Nanoparticles and Correlation with Their Physical Properties

**DOI:** 10.1155/2012/632698

**Published:** 2012-07-08

**Authors:** Ling Yien Ing, Noraziah Mohamad Zin, Atif Sarwar, Haliza Katas

**Affiliations:** ^1^Drug Delivery and Novel Targeting Research Group, Faculty of Pharmacy, Universiti Kebangsaan Malaysia, Kuala Lumpur Campus, Jalan Raja Muda Abdul Aziz, 50300 Kuala Lumpur, Malaysia; ^2^Novel Antibiotic Research Group, Faculty of Health Sciences, Universiti Kebangsaan Malaysia, Kuala Lumpur Campus, Jalan Raja Muda Abdul Aziz, 50300 Kuala Lumpur, Malaysia

## Abstract

The need of natural antimicrobials is paramount to avoid harmful synthetic chemicals. The study aimed to determine the antifungal activity of natural compound chitosan and its nanoparticles forms against *Candida albicans, Fusarium solani and Aspergillus niger*. Chitosan nanoparticles were prepared from low (LMW), high molecular weight (HMW) chitosan and its derivative, trimethyl chitosan (TMC). Particle size was increased when chitosan/TMC concentration was increased from 1 to 3 mg/mL. Their zeta potential ranged from +22 to +55 mV. Chitosan nanoparticles prepared from different concentrations of LMW and HMW were also found to serve a better inhibitory activity against *C*. *albicans* (MIC_LMW_ = 0.25–0.86 mg/mL and MIC_HMW_ = 0.6–1.0 mg/mL) and *F. solani* (MIC_LMW_ = 0.86–1.2 mg/mL and MIC_HMW_ = 0.5–1.2 mg/mL) compared to the solution form (MIC = 3 mg/mL for both MWs and species). This inhibitory effect was also influenced by particle size and zeta potential of chitosan nanoparticles. Besides, *Aspergillus niger* was found to be resistant to chitosan nanoparticles except for nanoparticles prepared from higher concentrations of HMW. Antifungal activity of nanoparticles prepared from TMC was negligible. The parent compound therefore could be formulated and applied as a natural antifungal agent into nanoparticles form to enhance its antifungal activity.

## 1. Introduction

For the past few decades, there has been a growing interest in the modification and application of chitosan in medical and health fields. Chitosan has been the material of choice for the preparation of nanoparticles in various applications due to its biodegradable and nontoxic properties. Chitosan is soluble in acidic condition and the free amino groups on its polymeric chains protonates and contributes to its positive charge [1]. Chitosan nanoparticles are formed spontaneously on the incorporation of polyanion such as tripolyphosphate (TPP) in chitosan solution under continuous stirring condition. These nanoparticles are then harvested and used for gene therapy and drug delivery applications [2, 3]. However, due to its poor solubility at pH above 6.5, various chitosan derivatives with enhanced water solubility are introduced through chemical modification process, for example, *N-*trimethyl chitosan (TMC).

Chitosan in its free polymer form has been proved to have antifungal activity against *Aspergillus niger*, *Alternaria alternata*, *Rhizopus oryzae*, *Phomopsis asparagi*, and *Rhizopus stolonifer* [4–6]. From these findings, it could be concluded that antifungal activity of chitosan was influenced by its molecular weight, degree of substitution, concentration, types of fungus, and types of functional groups in chitosan derivatives chains [6–10]. Basically, the antifungal activity is contributed by the polycationic nature of chitosan. Therefore, chitosan exhibits natural antifungal activity without the need of any chemical modification [6].

There are three mechanisms proposed as the inhibition mode of chitosan. In the first mechanism, plasma membrane of fungi is the main target of chitosan. The positive charge of chitosan enables it to interact with negatively charged phospholipid components of fungi membrane. This will increase the permeability of membrane and causes the leakage of cellular contents, which subsequently leads to cell death [11, 12]. For the second mechanism, chitosan acts as a chelating agent by binding to trace elements, causing the essential nutrients unavailable for normal growth of fungi [13]. Lastly, the third mechanism proposed that chitosan could penetrate cell wall of fungi and bind to its DNA. This will inhibit the synthesis of mRNA and, thus, affect the production of essential proteins and enzymes [14].

Currently, most of the research has focused on the antifungal activity of chitosan solution. Therefore, the main objective of this study was to investigate antifungal activity of chitosan nanoparticles and to determine its correlation with the physical characteristics of the nanoparticles particularly particle size and surface charge. In this study, *A. niger*, *F. solani*, and *C. albicans* were selected. Minimum inhibitory concentration (MIC_90_) of chitosan nanoparticles to inhibit the selected fungi was determined as it is used as an indicative measure for assessing antifungal activity of any compound.

## 2. Materials and Methods

### 2.1. Materials

Low molecular weight (LMW, MW = 70 kDa) chitosan (C_8_H_15_NO_6_)_*n*_  powder with 75–85% degree of deacetylation and high molecular weight chitosan (HMW, MW = 310 kDa) with 85% deacetylated were purchased from Sigma-Aldrich (Germany). *N-trimethyl chitosan* was obtained from Heppe Medical Chitosan GmbH (Germany). Pentasodium triphosphate, Na_5_P_3_O_10_  (TPP, M = 367.86 g/mol) and sodium hydroxide (NaOH, M = 39.9971 g/mol) were purchased from Merck kGaA (Germany). *Candida albicans, Aspergillus niger*, and *Fusarium solani* were pathogenic strain isolated from clinical specimens. Acetic acid glacial, CH_3_COOH (M = 60.05 g/mol) was obtained from R & M Chemicals (UK). All chemicals were of analytical grade and used as received.

### 2.2. Methods

#### 2.2.1. Preparation of Chitosan Solution

A concentration of 1.2% w/v chitosan, solution was prepared by dissolving 0.06 g of LMW and HMW chitosan in 5 mL of 2% v/v acetic acid solution. pH of the solution was later adjusted to 5.6 by adding sodium hydroxide solution to ensure acidic condition would not interfere with the antifungal determination [6]. TMC solution was prepared by dissolving 0.06 g TMC in 5 mL of distilled water.

#### 2.2.2. Preparation of Nanoparticles

LMW, HMW chitosan and TMC solution at concentration of 1, 2, and 3 mg/mL were prepared by dissolving 0.01, 0.02, and 0.03 g, respectively, of chitosan in 10 mL of 2% v/v acetic acid and distilled water (for TMC). In this study, nanoparticles were prepared by ionic gelation method via the interaction with TPP polyanion [15]. A volume of 1.2 mL of 0.1% w/v TPP solution was added to 3 mL of chitosan or TMC solution under continuous magnetic stirring at 700 rpm, and the nanoparticles were formed spontaneously. The particles were then incubated at room temperature for 30 minutes prior to further analysis. The resultant nanoparticles were then collected by centrifugation (Beckman Coulter Optima L-100XP Floor Centrifugation System) at 25000 rpm for 30 minutes. The supernatants were discarded, and the nanoparticles were redispersed in distilled water.

#### 2.2.3. Characterisation of Nanoparticles

Mean particle size (Z-average) and zeta potential of the nanoparticles were measured by using Malvern Zetasizer Nano ZS (UK). The measurements were performed at a temperature of 25°C in triplicate. Samples were appropriately diluted with distilled water prior to measurement. The values were reported as mean ± standard deviation. Nanoparticles morphology was examined by Philips Tecnai 12 Transmission Electron Microscope (TEM). The samples were stained using uranyl acetate and then analysed.

#### 2.2.4. Determination of Antifungal Activity

The antifungal activity of chitosan solution, nanoparticles of LMW, HMW chitosan, and TMC were tested on *C. albicans*, *A. niger*, and *F. solani*. Broth microdilution procedures were used with the reference of approved standard from Clinical and Laboratory Standard Institute [16, 17]. Potato dextrose agar and potato agar broth were used as medium. Amphotericin B and nontreated fungus were used as positive and negative control, respectively. Amphotericin B is a fungicidal agent that is widely used in treating serious systemic infections. Samples with the concentrations of 4 times higher than the desired concentration were prepared. After that, the samples were diluted 1 : 2 in potato dextrose broth medium by adding 0.05 mL of broth medium to 0.05 mL of samples. The working concentrations of antifungal solutions were prepared twofold higher than the desired concentration because the solutions would become a 1 : 2 dilution after the samples were mixed with inoculum. A volume of 0.1 mL of each antifungal solution was pipetted into different wells of 96-well microtiter plate. A series of dilution was done in order to determine the MIC_90_ of each sample. The inoculum suspensions of three different fungi were prepared. Each well was inoculated with 0.1 mL of corresponding inoculum suspension. Microtiter plates for *A. niger* and *F. solani* were incubated at room temperature, while for *C. albicans*, it was incubated at 37°C. At 48 hours following incubation, ocular density of each well in microtiter plate was examined by using microplate reader at 630 nm. The difference between ocular densities of each sample was compared with a negative control (without antifungal agent). Percentage of inhibition was calculated, and MIC_90_ was then determined.

#### 2.2.5. Statistical Analysis

Data were summarised as the mean ± standard deviation (SD). Data were analysed by using SPSS 17.0 with independent *t*-test, one-way ANOVA, or Pearson's correlation for normally distributed data. Nonparametric tests (Mann-Whitney test, Kruskal-Wallis test, and Spearman's correlation test) were used for nonnormal distributed data.

## 3. Results

### 3.1. Characterisation of Nanoparticles

#### 3.1.1. Particle Size and Zeta Potential before Centrifugation

The mean particle size for chitosan and TMC nanoparticles increased with the increasing concentration of chitosan or TMC and when a higher molecular weight was used (*P* < 0.05, Kruskal-Wallis test and one-way ANOVA). As summarized in Table 1, TMC generally produced the smallest nanoparticles, followed by LMW and HMW chitosan nanoparticles. However, at chitosan concentration of 1 mg/mL, TMC produced the largest nanoparticles compared to the others. All types of nanoparticles produced showed narrow size distributions with low PDI values (0.10–0.60) except for several formulations, HMW chitosan at 2 and 3 mg/mL. Besides, particle size of chitosan nanoparticles was found to be statistically correlated with chitosan molecular weight in which it increased when a higher molecular weight was used.

The mean zeta potential of chitosan nanoparticles is also presented in Table 1. According to the results obtained, higher values of zeta potential were obtained when HMW chitosan was used. Zeta potential was also found to be directly proportional to the concentration of chitosan or TMC used in the preparation of nanoparticles (*P* < 0.05, Kruskal-Wallis test). Higher concentrations of chitosan produced nanoparticles with higher values of zeta potential. In general, TMC nanoparticles had the lowest zeta potential, followed by LMW and HMW chitosan nanoparticles.

#### 3.1.2. Particle Size and Zeta Potential after Centrifugation

**Figure 1:**
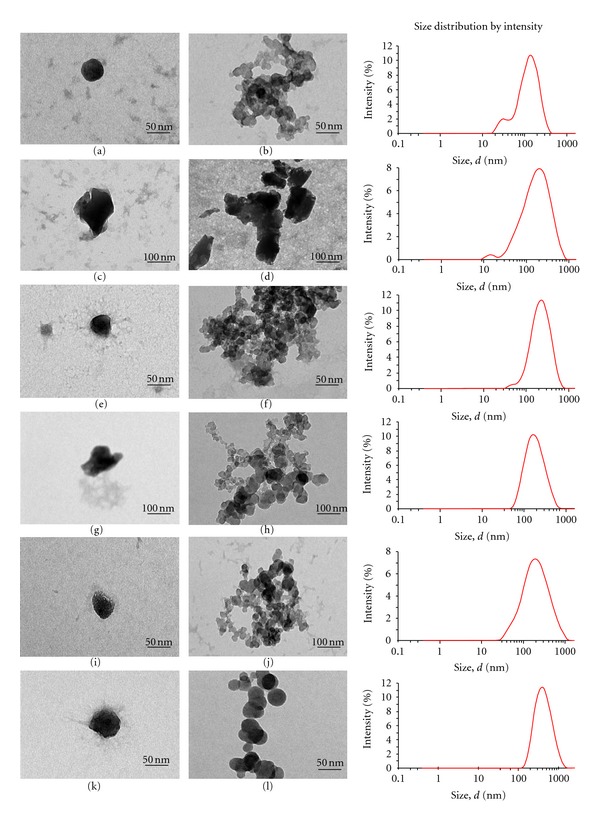
Top: TEM images and particle size distribution of LMW (a, b), HMW (c, d), and TMC (e, f) nanoparticles before centrifugation. Bottom: TEM images and particle size distribution of LMW (g, h), HMW (i, j), and TMC (k, l) nanoparticles after centrifugation. Nanoparticles were prepared from chitosan concentration of 1 mg/mL.

The mean particle size of nanoparticles after centrifugation is shown in Table 2. The mean particle size ranged from 170 to 435 nm. Generally, all nanoparticles were slightly larger in size after centrifugation except for LMW and HMW chitosan at concentration of 3 mg/mL which had smaller particle size. Despite increase in size, these nanoparticles had a relatively narrow particle size distribution with PDI values ranging from 0.2 to 0.6. Graphs for particle size distribution of chitosan nanoparticles before and after centrifugation are shown in Figure 1.

On the other hand, zeta potential of chitosan nanoparticles after centrifugation remained unchanged compared with the ones before centrifugation except for the TMC nanoparticles. The results also showed that zeta potential of these nanoparticles increased with higher molecular weight of chitosan.

 Morphology of different chitosan nanoparticles was investigated by using a TEM. The morphology of chitosan nanoparticles was found to be influenced by the type of chitosan used. TMC nanoparticles produced a more spherical particle compared to parent compound as depicted by Figure 1.

### 3.2. Antifungal Activities of Chitosan Nanoparticles

Table 3 shows the antifungal activities of chitosan solution and different types of chitosan nanoparticles. MIC_90_, or the minimum concentration of the sample that is needed to inhibit 90% of the fungus colonies [18], was used as a measurement for the antifungal activity of each nanoparticles sample. Any sample that had a smaller MIC value was considered to exhibit a stronger antifungal effect. Amphotericin B was used as a positive control. It was an effective antifungal agent with MIC_90_ as low as 0.002 mg/mL for *C. albicans *and *A. niger* while 0.02 mg/mL for *F. solani*. Chitosan, both in solution and nanoparticles forms, required a higher concentration to inhibit 90% of selected fungi species. Therefore, it indicated that natural antifungal activity of chitosan was not as strong as synthetic antifungal agent.

#### 3.2.1. *C. albicans*

LMW and HMW chitosan solution with MIC_90_ of 3 mg/mL was found to have less antifungal activity against *C. albicans* compared with chitosan nanoparticles. Among these nanoparticles, chitosan nanoparticles prepared from LMW chitosan at concentration of 1 mg/mL had the smallest particle size and showed the highest antifungal effect with MIC_90_ of 0.25 mg/mL. Antifungal activities of chitosan nanoparticles were shown to be independent of chitosan molecular weight as MIC_90_ of chitosan nanoparticles made from LMW and HMW did not show significant difference, except when the nanoparticles were prepared at low concentration (1 mg/mL). Furthermore, a correlation between particle size of the same MW chitosan nanoparticles and MIC_90_ was statistically proven. The inhibitory activity of chitosan nanoparticles against *C. albicans* increased with the decreasing size of the LMW chitosan nanoparticles (Pearson's correlation coefficient: +0.528). In contrast to that, an inverse relationship was observed for HMW chitosan nanoparticles.

#### 3.2.2. *F. solani*

Similar to *C. albicans*, chitosan nanoparticles had better inhibitory effects against *F. solani* compared to solution form (*P* < 0.05, Kruskal-Wallis analysis). In contrast to *C. albicans*, *F. solani *was found to be more susceptible to inhibitory effect of HMW chitosan nanoparticles. The highest activity was obtained with the smallest HMW chitosan nanoparticles (chitosan concentration of 1 mg/mL). For other particle sizes, (chitosan concentration of 2 and 3 mg/mL), antifungal effect was found to be similar between LMW and HMW. Unlike other types of chitosan nanoparticles, TMC nanoparticles had no inhibitory activity against *F. solani. *Particle size of chitosan nanoparticles was statistically correlated with antifungal activity towards *F. solani* (Pearson's correlation coefficient: 0.528) when comparing with the same MW of chitosan.

#### 3.2.3. *A. niger*

The data obtained suggested that *A. niger* resisted more to antifungal effect of chitosan compared with *F. solani* and *C. albicans*. Inhibitory activity could only be detected for chitosan solution (LMW and HMW) and chitosan nanoparticles prepared from higher concentrations of HMW chitosan (2 and 3 mg/mL). Other nanoparticles had negligible inhibitory effect against *A. niger*.

## 4. Discussions

Chitosan or TMC nanoparticles can be prepared using many methods such as ionic gelation, complex coacervation, emulsion cross-linking, and spray drying. In this study, ionic gelation method was applied because the method is easy and fast to be carried out [19]. This simple technique involves electrostatic interaction between positively charged amino group of chitosan and negatively charged polyanions. Formation of nanoparticles occurs spontaneously through the formation of intra- and intermolecular cross-linkages under a constant stirring at ambient temperature. Besides that, this method is highly controllable, and, thus, important properties of nanoparticles such as particle size or surface charge can be easily manipulated by changing parameters such as concentration of chitosan, chitosan-to-polyanion weight ratio, and solution pH [20].

Particle size and zeta potential are the important properties which may influence the antifungal activity of nanoparticles. Nanoparticles with different particle size or zeta potential may have different mechanisms of inhibition against fungi. Therefore, in this study, the influence of particle size and zeta potential on antifungal effect was studied on *C. albicans*, *F. solani*, and *A. niger* by using nanoparticle samples with different particle size and zeta potential. There are several factors that affect particle size of nanoparticles. This includes concentration and molecular weight of chitosan [20]. In this study, the effects of different concentrations and molecular weights on particle size of chitosan or TMC nanoparticles were investigated. The results showed that the size of nanoparticles, especially HMW chitosan nanoparticles, was greatly influenced by the concentration of chitosan which was added into a constant amount of TPP. A linear relationship was also observed where increase in concentration would increase particle size. Similar relationship was also observed with the molecular weight of chitosan in which the effect on particle size was also very prominent. These linear relationships enable easy manipulation of nanoparticle size for application in different fields.

A smaller particle size with a lower concentration or molecular weight was expected to be due to the decreased viscosity which led to better solubility of chitosan in distilled water or acetic acid solution. Hence, more amino groups on chitosan or TMC would be protonated. This would allow for more efficient interaction between negatively charged chitosan and polyanion [21]. TMC nanoparticles have smaller particle size than LMW and HMW chitosan nanoparticles, except for chitosan concentration at 1 mg/mL. Higher charge density of TMC than chitosan molecule was expected attributed to the results. The high charge density of TMC resulted in stronger electrostatic interactions with the TPP and allowed more TPP to interact with the polymer [22]. However, the cause of obtaining larger particle size for TMC concentration of 1 mg/mL is currently unclear. Gan et al. [20] reported that low surface charge on nanoparticles causes decreasing in electrostatic repulsion between particles and hence increases the probability of particle aggregation. Nanoparticles with surface charge of +30 mV had been shown to be stable as the surface charge is sufficient to prevent aggregation of the particles [23]. Therefore, these could be the reasons to explain the largest size of TMC nanoparticles when prepared from the lowest concentration (1 mg/mL) as they had zeta potential of around +20 mV. Furthermore, most of the samples showed narrow size distribution except for nanoparticles made from HMW chitosan at higher concentrations (2 and 3 mg/mL). This was expected to be due to the solubility property of HMW chitosan which is less soluble than LMW chitosan and therefore produced nanoparticles with different sizes.

Zeta potential has been suggested as a key factor contributing to antifungal effect of chitosan through the interaction with negatively charged microbial surface [24]. In this study, zeta potential of chitosan or TMC nanoparticles showed a net positive surface charge due to excess positive charge of chitosan or TMC molecules after interaction with TPP. The results obtained proved that the magnitude of particle positive charge increased linearly with the increasing concentration or molecular weight. This was expected due to the increase in positive charge available to interact with negatively charged TPP as the amount of TPP was constant [21]. According to Tables 1 and 2, all TMC nanoparticles had the lowest value of zeta potential. This finding differed from the reported study by Boonyo et al. [25] which claimed that TMC nanoparticles should have a higher zeta potential than chitosan nanoparticles due to the presence of permanently positive charged sites in TMC chains.

Ultracentrifugation technique was used to wash and harvest nanoparticles produced. In this study, some types of nanoparticles showed increasing or decreasing in particle size after being subjected to ultracentrifugation. This was expected to be due to the technique that works at high-speed principle which causes particles to aggregate or loss of chitosan molecules from the main networks of chitosan and TPP particles which resulted in increased or decreased particle size [26]. For example, in the case of HMW, particle size of nanoparticles prepared from 3 mg/mL significantly reduced from 1265 ± 206.48 to 301 ± 72.85 nm after centrifugation. The results could be explained by the reason that smaller particles may adsorb on the surface of larger particles via partial physical interactions to form agglomerates. This could be observed from its high PDI value (0.99 ± 0.02). It indicated that the particle size was widely distributed before centrifugation. However, when these particles were centrifuged, the surface adsorbed particles were washed away from the larger particles due to high centrifugation speed. On the other hand, nanoparticles were considered as stable if their particle size before and after ultracentrifugation remained unchanged.

Chitosan has been proven to have antifungal activity, and therefore it has attracted a great attention from many researchers. In the present study, the antifungal activity of chitosan solution and nanoparticles was studied. Previous studies showed that the effectiveness of chitosan did not depend solely on the chitosan formulation but also on the type of fungus. The relationship between particle size or zeta potential on antifungal activity was therefore studied against three different species of fungi. *C. albicans* is a fungus that infects human skin as well as mucous membrane. It may enter into blood stream and spread throughout the body [7, 27]. *Fusarium* species, on the other hand, are frequently reported as the causative agent in opportunistic infections in human [28]. *A. niger* is the most common causative agent encountered in food contamination cases. Although it is not a common human pathogen, in high concentration, it may cause aspergillosis [29].

Based on the results obtained, chitosan solution showed higher MIC_90_ values compared with nanoparticles for the selected fungi species. This therefore suggested that chitosan solution was less effective as an antifungal agent compared with LMW and HMW chitosan nanoparticles. This finding coincides with the previous reported study by Qi et al. [30] which demonstrated that chitosan nanoparticles exhibited higher antimicrobial activity due to their special characters of the nanoparticles such as small and compact particle as well as high surface charge. This could be explained by the fact that the negatively charged plasma membrane is the main target site of polycation [31]. Therefore, the polycationic chitosan nanoparticles with high surface charge will interact more effectively with the fungus compared with free form of chitosan polymer. Furthermore, chitosan nanoparticles have a higher affinity to bind to fungal cells. Nanosized chitosan nanoparticles contribute to a larger surface area and cause nanoparticles to be able to adsorb more tightly onto the surface of fungal cells and disrupt the membrane integrity [30]. A study carried out by Ma and Lim [32] reported that cellular uptake of chitosan nanoparticles into cells was higher than that of chitosan molecules as the bulk chitosan molecules were located extracellularly. This suggested that chitosan nanoparticles might be able to diffuse into fungal cell and hence disrupt the synthesis of DNA as well as RNA. This could explain a better antifungal activity of chitosan nanoparticles compared to its free polymer or solution form.

In current study, TMC has been used as it is soluble in water, and it is paramount to investigate water solubility property on the antifungal activity. TMC nanoparticles, however, had shown to exert no antifungal activity against the selected fungi. Recent research has proved that chitosan derivatives had weak or no antimicrobial activity although they are highly water-soluble [33–35]. A better antifungal activity by the parent compound was correlated with that of water insolubility of chitosan which precipitates and stacks on the microbial cell surface as the physiological pH in microbial cells is around neutral. The formation of impermeable layer will block the channels on the cell surface and hence prevent the transportation of essential nutrients which are crucial for survival of microbial cells. Contrary to that, the water soluble chitosan derivatives are unable to form such layer, and therefore they exert no antimicrobial activity.

All LMW and HMW chitosan nanoparticles could inhibit the growth of *C. albicans*. The smallest LMW chitosan nanoparticles exerted the highest anticandidal activity. Tayel et al. [7] also reported that LMW chitosan was more effective against *C. albicans* than other types. *C. albicans* was more susceptible to be inhibited by chitosan nanoparticles if compared with other types of fungi. This could be due to the presence of anionic charged sialic acid in cell wall constituent [36]. Particle size was also found to have influence on the inhibition of *C. albicans* in the present study. For LMW, smaller nanoparticles had stronger antifungal effect. This finding was in agreement with other study which reported that with a decrease in the size of silver and titanium nanoparticles from 29 nm to 20–25 nm, their antimicrobial activity increased significantly [37]. The size of particles plays an important role in determination of antimicrobial activity of nanoparticles as they enter the cell walls of microbes through carrier proteins or ion channel. Therefore, smaller particle size will result in a better uptake of nanoparticles into microbial cell [38]. The proposed inhibition mechanism of chitosan nanoparticles against *C. albicans* was therefore expected to be through diffusion of nanoparticles into the fungal cells, followed by inhibition of DNA or RNA synthesis, subsequently causing a direct cell death. In case of HMW, anticandidal activity was observed to increase as the particle size increased. The results could be explained with the fact that these nanoparticles had high particle surface charges of about +50–54 mV. Particle surface charge plays a role in the inhibitory effect of chitosan nanoparticles by contributing a positive charge to improve the interaction between nanoparticles and negatively charged microbial cell surface [39]. This in turn alters fungi cell membrane permeability which eventually induces leakage of intracellular material. This coincides with the previous reported study which showed that chitosan particles would only inhibit microbial growth when they were positively charged [40].

Chitosan has found to interfere with the growth of *F. solani* [41–43]. In the present study, the smallest HMW chitosan nanoparticles showed a better antifungal activity against *F. solani* compared with all other nanoparticles. Similar finding was also reported by Kendra and Hadwiger [42]. Particle size and surface charge of nanoparticles were found to be statistically correlated with their MIC_90_. Their fungal inhibitory activity increases as the particle size and zeta potential decreases. In this regard, particle size of chitosan nanoparticles may have superior influence on the antifungal activity towards *F. solani* than their surface charge.

In contrast, *A. niger* was found to be highly resistant to chitosan. Only chitosan solution and nanoparticles prepared at high concentration of HMW chitosan were able to inhibit the growth of this fungal. This finding also coincides with another reported study by Ziani et al. [6] which demonstrated that HMW chitosan was more effective to inhibit *A. niger*. According to Allan and Hadwiger [44], fungi that have chitosan as one of the components in the cell wall are more resistant to externally amended chitosan. This fact could therefore explain the high resistance of *A. niger* as it contains 10% of chitin in its cell wall [45].

 The findings from this study may differ from some other previous reported studies due to the differences in experimental conditions. Further investigation on different species of fungi is being carried out because type of fungi is also affecting antifungal activity of chitosan. Besides, more chitosan derivatives are involved in this ongoing study.

## 5. Conclusions

A linear relationship between molecular weight and particle size/zeta potential was statistically proven. This provided a platform for easy manipulation of physicochemical properties of nanoparticles suitable for their intended application. Formulation of chitosan into nanoparticles form was found to increase its antifungal effect significantly. Therefore, it is anticipated that chitosan nanoparticles have the potential of becoming a powerful and safe natural antifungal agent.

## Figures and Tables

**Table 1:** Mean particle size, PDI, and zeta potential of different concentrations (mg/mL) for chitosan and TMC nanoparticles with constant amount of 0.1% w/v TPP before centrifugation, *n* = 3.

	Chitosan concentration	Particle size (nm)	PDI	Zeta potential (mV)
	(mg/mL)	(Mean ± SD)	(Mean ± SD)	(Mean ± SD)
LMW	1	101 ± 9.58^∗^	0.366 ± 0.047	+35 ± 6.53^∗^
	2	169 ± 13.47^∗^	0.453 ± 0.018	+43 ± 2.05^∗^
	3	348 ± 35.74^∗^	0.594 ± 0.121	+47 ± 4.37^∗^

HMW	1	136 ± 8.64^∗^	0.378 ± 0.073	+38 ± 1.68^∗^
	2	276 ± 46.77^∗^	0.792 ± 0.167	+50 ± 1.79^∗^
	3	1265 ± 206.48^∗^	0.990 ± 0.021	+55 ± 3.46^∗^

TMC	1	191 ± 21.22^∗^	0.155 ± 0.095	+22 ± 2.41^∗^
	2	159 ± 3.00^∗^	0.192 ± 0.032	+28 ± 3.23^∗^
	3	212 ± 7.31^∗^	0.263 ± 0.030	+29 ± 4.33^∗^

^∗^Significantly different (*P* < 0.001) between groups for each concentration.

**Table 2:** Mean particle size, PDI, and zeta potential of different concentration (mg/mL) for chitosan and TMC nanoparticles with constant amount of 0.1% w/v TPP after centrifugation, *n* = 3.

	Chitosan concentration	Particle size (nm)	PDI	Zeta potential (mV)
	(mg/mL)	(Mean ± SD)	(Mean ± SD)	(Mean ± SD)
LMW	1	174 ± 38.47^∗^	0.457 ± 0.115	+39 ± 8.56
	2	233 ± 41.38	0.377 ± 0.093	+38 ± 1.85^∗^
	3	255 ± 42.81	0.510 ± 0.104	+48 ± 4.78^∗^

HMW	1	210 ± 24.54^∗^	0.532 ± 0.192	+40 ± 3.16
	2	263 ± 86.44	0.551 ± 0.185	+52 ± 6.27^∗^
	3	301 ± 72.85	0.566 ± 0.176	+54 ± 5.01^∗^

TMC	1	433 ± 79.59^∗^	0.513 ± 0.123	+37 ± 2.75
	2	211 ± 89.26	0.448 ± 0.190	+33 ± 4.79^∗^
	3	297 ± 64.72	0.243 ± 0.073	+37 ± 2.52^∗^

^∗^Significantly different between groups (*P* < 0.001) for each concentration.

**Table 3:** Antifungal activity of chitosan solution and nanoparticles against selected fungi species, *n* = 3. CS: chitosan; NP: nanoparticles.

Sample		Particle size	Zeta potential		MIC_90_ (mg/mL)	
		(nm)	(mV)	*C. albicans*	*F. solani*	*A. niger*
Amphotericin B (positive control)		—	—	0.002	0.02	0.002
HMW CS solution		—	—	3	3	3
LMW CS solution				3	3	3
TMC solution				—	—	—

	LMW	174 ± 38.47	+39 ± 8.56	**0.25**	1	—
CS NP prepared from 1 mg/mL CS	HMW	210 ± 24.54	+40 ± 3.16	1	**0.5**	—
	TMC	433 ± 79.59	+37 ± 2.75	—	—	—

	LMW	233 ± 41.38	+38 ± 1.85	0.8572	0.8572	—
Cs NP prepared from 2 mg/mL CS	HMW	263 ± 86.44	+52 ± 6.27	0.8572	0.8572	**1.7143**
	TMC	211 ± 89.26	+33 ± 4.79	—	—	—

	LMW	255 ± 42.81	+48 ± 4.78	0.6072	1.2143	—
Cs NP prepared from 3 mg/mL CS	HMW	301 ± 72.85	+54 ± 5.01	0.6072	1.2143	2.4286
	TMC	297 ± 64.72	+37 ± 2.52	—	—	—
